# A microRNA profile associated with *Opisthorchis viverrini*-induced cholangiocarcinoma in tissue and plasma

**DOI:** 10.1186/s12885-015-1270-5

**Published:** 2015-04-23

**Authors:** Jordan Plieskatt, Gabriel Rinaldi, Yanjun Feng, Jin Peng, Samantha Easley, Xinying Jia, Jeremy Potriquet, Chawalit Pairojkul, Vajarabhongsa Bhudhisawasdi, Banchob Sripa, Paul J Brindley, Jeffrey Bethony, Jason Mulvenna

**Affiliations:** 1 Department of Microbiology, Immunology and Tropical Medicine, School of Medicine and Health Sciences, George Washington University, Washington, DC 20037 USA; 2 Research Center for Neglected Diseases of Poverty, School of Medicine and Health Sciences, George Washington University, Washington, DC 20037 USA; 3 Department of Pathology, School of Medicine and Health Sciences, George Washington University, Washington, DC 20037 USA; 4 QIMR Berghofer Medical Research Institute, Infectious Disease and Cancer, Brisbane, Queensland 4006 Australia; 5 Faculty of Medicine, Khon Kaen University, Khon Kaen, 40002 Thailand; 6 The University of Queensland, School of Biomedical Sciences, Brisbane, Queensland 4072 Australia

**Keywords:** MicroRNA, Cholangiocarcinoma, Intrahepatic cholangiocarcinoma, Opisthorchis viverrini, RNA-seq

## Abstract

**Background:**

Intrahepatic cholangiocarcinoma (ICC) is a highly aggressive tumor of the bile duct, and a significant public health problem in East Asia, where it is associated with infection by the parasite *Opisthorchis viverrini*. ICC is often detected at an advanced stage and with a poor prognosis, making a biomarker for early detection a priority.

**Methods:**

We have comprehensively profiled miRNA expression levels in ICC tumor tissue using small RNA-Seq and validated these profiles using quantitative PCR on matched plasma samples.

**Results:**

Distinct miRNA profiles were associated with increasing histological differentiation of ICC tumor tissue. We also observed that histologically normal tissue adjacent to ICC tumor displayed miRNA expression profiles more similar to tumor than liver tissue from healthy donors. In plasma samples, an eight-miRNA signature associated with ICC, regardless of the degree of histological differentiation of its matched tissue, forming the basis of a circulating miRNA-based biomarker for ICC.

**Conclusions:**

The association of unique miRNA profiles with different ICC subtypes suggests the involvement of specific miRNAs during ICC tumor progression. In plasma, an eight-miRNA signature associated with ICC could form the foundation of an accessible (plasma-based) miRNA-based biomarker for the early detection of ICC.

**Electronic supplementary material:**

The online version of this article (doi:10.1186/s12885-015-1270-5) contains supplementary material, which is available to authorized users.

## Background

Intrahepatic cholangiocarcinoma (ICC) is an aggressive subtype of bile duct cancer, which arises in the cholangiocytes of the biliary ducts that extend into the upper hepatoduodenal ligament. While ICC is rare in developed countries such the United States (0.5 per 100,000), ICC is a significant public health problem in low and middle-income countries (LMICs) of Southeast Asia (incidence of 96 per 100,000), particularly the Mekong River Basin countries of Thailand, Laos, Cambodia, and Vietnam [1-3]. This variation in incidence reflects the different underlying etiologies of ICC. In the Mekong River Basin, ICC is strongly associated with chronic infection by the food-borne liver fluke *Opisthorchis viverrini* (Ov) [4]: one of only three eukaryote pathogens considered Group 1 carcinogens [4]. Ov is a ribbon-like, two-centimeter long parasite that is acquired by eating under-cooked cyprinoid fish that harbor the metacercarial stage of this parasite [2]. Upon ingestion, the metacercariae excyst in the host duodenum and migrate up the biliary tree, inhabiting the host bile ducts for years (even decades), feeding on epithelial cells of the biliary tract. This prolonged injury to the bile duct epithelia creates a persistent “smouldering inflammatory milieu” [5], that eventually results in several hepatobiliary abnormalities, principal among them ICC [5].

The location of ICC tumors in the upper hepatoduodenal ligament makes this tumor asymptomatic and hence difficult to detect in early stages. Moreover, its location in the upper hepatoduodenal ligament increases the opportunities for distant metastasis due to the proximity to the lymphatic and vascular systems of the liver [6]. As such, these slow-growing tumors are usually diagnosed at an advanced stage, when the primary cancer is no longer amenable to surgical extirpation and has metastasized to other organs [5]. The median survival rate of Ov-induced ICC is less than 24 months [7]. This poor prognosis highlights the need for diagnostic biomarkers of Ov-induced ICC, especially in resource poor areas, where the incidence is highest and access to health care is difficult.

Over the last five years, microRNAs (miRNAs) have become key biomarker candidates for carcinogenesis as they play a role in numerous physiological and pathological processes, including cellular transformation, tumor differentiation, neoplastic proliferation, and apoptosis [8]. In cholangiocarcinoma, a growing number of miRNAs have been associated with the disease and a functional role has been defined for many of these (for examples see [9] and [10]; also reviewed in [11] and summarized in Table 1). MicroRNAs are very stable small non-coding RNA species and hence well preserved in formalin fixed paraffin embedded (FFPE) tumor blocks, an ample sample source, considered unsuitable for transcriptome studies. Recently, we reported the first comprehensive microarray-based profiling of miRNA expression using FFPE from the three most common subtypes of Ov-induced ICC tumors [12]: moderately differentiated ICC, papillary type ICC, and well-differentiated ICC. Each Ov-induced ICC subtype exhibited a distinct miRNA profile, which suggested the involvement of specific sets of miRNAs in the progression of this tumor.

**Table 1:** **Comparison of dysregulated miRNAs associated with ICC to those reported in the literature**

miRNA	Function	Target	Direction this work	Tissue	Ref
**Up-regulated in the literature**					
Let-7a	Cell survival	NF2	-	Cell lines	[50]
miR-21	Apoptosis, proliferation,	MBD2, 15-PGDH/HPGD,	Up	Cell lines, Tissue	[10,51-54]
	invasion, metastasis	PTEN,PDCD4, TIMP3			
miR-25	Apoptosis	DR4	Up	Cell lines, Tissue	[55]
			(CCT v. N-NT)		
miR-26a	Proliferation, colony formation,	GSK-3	Down	Cell lines, Tissue	[56]
	tumor growth		(CCT v. N-NT)		
miR-29b	-	-	Up	Tissue	[57]
			(Pap. v. N-NT)		
miR-31	Proliferation, apoptosis	RASA1	Up	Cell lines, Tissue	[58]
miR-34b	-	-	Up	Tissue	[57]
miR-135	-	-	Up	Tissue	[57]
miR-141	Proliferation, circadian rhythm	CLOCK	Up	Cell lines	[51]
miR-146a	-	-	Up	Tissue	[57]
			(Pap. v. N-NT)		
miR-192	-	-	Down	Tissue	[57]
			(CCT v. N-NT)		
miR-194	-	-	-	Tissue	[57]
miR-200a	Chemoresistance	PTPN12	Up	Cell lines	[57]
miR-200b	Chemoresistance	PTPN12	Up	Cell lines	[51,57]
miR-200c	Chemoresistance	PTPN12	Up	Cell lines	[57]
miR-203	-	-	Up	Tissue	[57]
miR-210	Proliferation	Mnt	Up	Mouse tissue	[59]
			(CCT v. N-NT)		
miR-215	-	-	-	Tissue	[57]
miR-221	-	-	Up	Tissue	[57]
			(CCT v. N-NT)		
miR-361	-	-	Up	Tissue	[57]
			(CCT v. N-NT)		
miR-375	-	-	Up	Tissue	[57]
			(CCT v. N-NT)		
miR-421	Proliferation, migration,	FXR	Up	Cell lines, Tissue	[60]
	colony formation		(CCT v. N-NT)		
miR-429	-	-	Up	Tissue	[57]
miR-582	-	-	-	Tissue	[57]
miR-892b	-	-	Up	Tissue	[57]
**Down-regulated in the literature**					
miR-29b	Gemcitabine sensitivity, apoptosis	PIK3R1, MMP-2, Mcl1	-	Cell lines	[61,62]
miR-34a	Cell cycle, proliferation	c-Myc	Up	Mouse tissue	[59]
miR-124	Migration, invasion	SMYD3	-	Cell lines	[63]
miR-138	Proliferation, cell cycle,	RhoC	Up	Tissue	[64]
	migration, invasion				
miR-144	Proliferation, invasion	Pafah1b2	Down	Tissue	[57]
miR-148a	Proliferation	DNMT-1	Down	Cell lines	[65]
miR-200b/c	Migration, invasion	Rho-kinase2, SUZ12	Up	Tissue	[66]
miR-204	EMT, migration,	Slug, Bcl-2	Down	Cell lines, Tissue	[67,68]
	invasion, apoptosis		(Pap. v. N-NT)		
miR-214	EMT, metastasis	Twist	Up	Tissue	[69]
			(CCT v. N-NT)		
miR-320	Apoptosis	Mcl-1	Down	Cell lines, Tissue	[68]
			(CCT v. N-NT)		
miR-370	Proliferation	MAP3K8	Down*	Cell lines	[70]
miR-373	Epigenetics	MBD2	-	Tissue	[71]
miR-376c	Migration	GRB2	Down	Cell lines	[72]
			(CCT v. N-NT)		
miR-451	-	-	Down	Tissue	[57]
miR-486	-	-	Down	Tissue	[57]
miR-494	Proliferation, cell cycle	CDK6	-	Cell lines	[73]
miR-495	-	-	Down	Tissue	[57]
			(Pap. v. N-NT)		
miR-513	-	-	-	Tissue	[57]
miR-625	-	-	Up	Tissue	[57]
			(CCT v. N-NT)		
miR-1926	-	-	-	Tissue	[57]

Unless otherwise stated ‘Direction this work’ refers to the CCT v. D-NT comparison.

In the current manuscript, we confirm and extend these findings using small-RNA Next Generation Sequencing (NGS). In addition we verified if tissue-based miRNA profiles were also detectable as circulating miRNA (c-miRNA) in matched plasma samples, a more accessible biomarker source than tissue. MicroRNAs in the blood circulate as signaling molecules during carcinogenesis [13-17], are “stable, reproducible, and consistent among individuals with the same cancer” [18] and hence have already been used as circulating biomarkers for breast [19], colorectal [20] and ovarian cancers [21]. While most studies of miRNA expression in cancer have focused on biomarker discovery in either tumor tissue or blood (i.e., serum or plasma), our study is among the first to compare different sample matrices (tissue and blood) for biomarker discovery by using paired samples (i.e., tissue and plasma from the same case), using two different discovery methods (microarray and small RNA-Seq). Hence, not only does the current manuscript inform our current basic understanding of miRNA in Ov-induced ICC, it also provides a methodological advance by following a biomarker discovery pipeline that starts with tissue-based biomarker discovery and then verifies candidate biomarkers in the blood.

## Methods

### Study Samples: tissue and matched plasma

FFPE liver sections and matched plasma samples from histologically confirmed Ov-induced ICC patients archived at the Liver Fluke and Cholangiocarcinoma Research Center, Faculty of Medicine, Khon Kaen University, Thailand were studied. The 14 tumor samples were derived from liver resections performed in the course of palliative treatment for confirmed cases of Ov-associated ICC at the Khon Kaen University’s Srinagarind Hospital, Khon Kaen, Thailand and are referred to as cholangiocarcinoma tissue (CTT). In addition, non-tumor tissue, microdissected distal from any observed dysplasia or frank carcinoma from the same CTT tumor block as noted above, were also examined and are referred to as Distal Non-Tumor (D-NT) tissue. Finally, non-tumor FFPE controls derived from liver biopsies of nine individuals suspected of severe steatosis or steatohepatitis prior to gastric bypass surgery were used to assess baseline liver histology of individual from non Ov endemic areas (USA) and are referred to as Normal Non-Tumor tissue (N-NT). The nine control individuals (N-NT) were female with an average age of 45 years (95% Confidence Interval of 38 to 54 years of age). Detailed clinico-pathological information and representative images of the tissues used in the current study are presented in detail in the previous manuscript, in which tissue-based miRNAs were assessed by microarray [12].

The ICC plasma samples included the following samples matched from the tissue based studies described above: four plasma matched to the well differentiated ICC tumor tissue, two plasma matched to the moderately differentiated ICC tissue, and six plasma matched to papillary graded tumors (Table 2). All but two plasma samples, B091 and Y070 (Table 2), were matched to tissue samples used in RNA-Seq analysis. Nine control plasma from individuals not resident in an Ov endemic area (USA) were utilized in quantitative PCR (qPCR) analysis alone as non-endemic controls.

**Table 2:** **Histological gradings of samples used for RNA-Seq and qPCR analysis of miRNA expression profiles**

ID	Sex	Age	Histological grade^a^	Gross classification	Microarray analysis[12]	RNA-Seq analysis	Paired plasma analysis (qPCR)
B070	M	61	WD	Mass-forming	X	X	
B079	M	61	WD	Periductal infiltrating, invasive intraductal and mixed	X	X	X
B083	F	53	WD	Mass-forming	X	X	X
B090	M	58	WD	Mass-forming	X	X	X
B099	M	48	WD	Mass-forming	X	X	X
Y042	M	61	WD	Mass-forming	X	X	X
B091	M	63	MD	Periductal infiltrating, invasive intraductal and mixed	X		X
Y070	F	63	MD	Mass forming	X		X
Y056	F	56	PC	Periductal infiltrating, invasive intraductal and mixed	X	X	X
Y062	M	57	PC	Periductal infiltrating, invasive intraductal and mixed	X	X	
B040	M	64	PC	Mass forming	X	X	X
Y083	F	51	PC	Mass forming	X	X	X
Y088	F	58	PC	Periductal infiltrating, invasive intraductal and mixed	X	X	X
Y089	F	60	PC	Mass forming	X	X	
Y093	M	63	PC	Periductal infiltrating, invasive intraductal and mixed	X	X	X
Y096	F	64	PC	Mass forming	X	X	X

^a^Histological types: tumor differentiation: WD = Well Differentiated tubular adenocarcinoma; MD = Moderately Differentiated tubular adenocarcinoma; and PC = Papillary Carcinoma.

Samples were further annotated including TNM anud staging in [12].

The Human Research Ethics Committee, Khon Kaen University, approved the study protocols for obtaining the human liver samples (HE571294) and both the Khon Kaen University and George Washington University IRBs determined that the samples used in this study did not meet the definition of human subjects research; i.e., a living individual about whom an investigator conducting research obtains: a) data through intervention or interaction with the individual or b) private identifiable information. This determination was made since the samples were limited to preexisting, de-identified specimen analysis labeled with a random code.

### Histological grading

Histological grading was done as described by the International Agency for Research on Cancer (IARC) [22]. In brief, assignment of the histological grade of well-differentiated adenocarcinoma to a tumor sample required that 95% of the tumor contain glands. For moderately differentiated ICC, tissue was required to have between 40 to 94% of the tumor composed of glands [22]. Though neither poorly differentiated nor undifferentiated carcinomas were used in this study, they would have had to display between 5 to 39% of the tumor containing glands or less than 5% of glandular structures, respectively [22]. In the case of papillary ICC, we again followed the IARC classification for tumors of the gallbladder and extrahepatic bile ducts [22], with the lesions having to consist predominantly of papillary structures lined by cells with a biliary phenotype, with good demarcation and consisting of papillary structures lined by tall columnar cells [22].

### RNA isolation from FFPE

RNA used was previously isolated from the dissected FFPE sections using the miRNeasy FFPE kit (Qiagen) [12] according to the manufacturer’s protocol and as previously described [23]. Total RNA was eluted in a volume of 30 μL RNase-free water. Concentration, purity and integrity for the RNA were determined by spectrophotometry (Nanodrop 1000) and Agilent 2100 Bioanalyzer/Agilent RNA 6000 Nano Kit and Agilent Small RNA kit. Purified RNA was stored at < −50°C.

### RNA isolation from matched plasma

RNA was isolated from plasma using the miRNeasy Serum/Plasma kit (Qiagen) according to manufacturer’s protocol. Briefly, 1 mL QIAzol lysis reagent was added to 200 μL thawed plasma, mixed and incubated at room temperature for 5 minutes. As a miRNA mimic, 3.5 μL of Spike-In Control (at 1.6 × 108 copies/μL of cel-miR-39-3p was added in addition to 200 μL chloroform (Fisher). Following shaking, incubation and centrifugation, the upper aqueous phase was transferred and 900 μL ethanol (Acros Chemical) was added and transferred to the RNeasy MinElute column. The column was washed with RWT, RPE, and 80% Ethanol (Acros Chemical), followed by drying and eluted in 14 μL RNase-free water. The concentration, purity and integrity were analyzed and stored as described above.

### Microarray analysis

Microarray analysis using the Agilent human miRNA microarray (miRBase Release 16.0) of the FFPE cases is extensively described in our previous manuscript [12] and the data was used here to compare the results of the two discovery platform microarray and small RNA-Seq data comparison.

### Small RNA sequencing

RNA purified from FFPE samples were depleted of rRNA by treatment with the Ribo-Zero rRNA Removal Kit (Cat. No. RZH1086, Epicentre), as described by the manufacturer. Briefly, biotinylated capture probes directed against rRNA sequences were added to total RNA samples and allowed to hybridize. Biotinylated complexes were removed using streptavidin-conjugated microbeads and non-ribosomal RNAs precipitated in ethanol. Libraries for small RNA sequencing were prepared using the TruSeq Small RNA Sample Prep Kit (Illumina). Illumina libraries were constructed from 1,000 ng of total RNA. Briefly, indexed oligonucleotide adapters were ligated to both the 3’-hydroxyl end and the 5’-phosphate end of the miRNAs using T4 RNA Ligase (New England Biolabs). RNA was reverse-transcribed and amplified using 14 cycles of PCR with primers targeting the 5’- and 3’- adapters, a specific index sequence, and Illumina sequencing adapters. The resulting products were analyzed and quantified using Agilent 2100 BioAnalyzer and the mole amount of mature miRNA present in the library was estimated by integrating the area under the curve in the 145–160 bp range. Individual libraries were mixed to create multiplexed pools, the mixture was gel purified, and the 145–160 bp range of RNA excised from the gel, crushed using a Gel Breaker tube (IST Engineering), eluted with nuclease-free water, and precipitated in ethanol. The concentration of the final library pool was determined using the PicoGreen system (Invitrogen) and the size distribution of the pool by the Agilent 2100 Bioanalyzer. Library pools were normalized to 2 nM for sequencing. Sequencing was performed using an Illumina Genome Analyzer IIx. Library preparation and small RNA sequencing was performed by Expression Analysis, A Quintiles Company (Durham, NC).

### MicroRNA alignment, mapping and annotation

Adapter sequences were clipped from deep sequencing reads using FastqMcf (http://code.google.com/p/ea-utils/wiki/FastqMcf and initial quality assessment performed using FastQC (http://www.bioinformatics.babraham.ac.uk/projects/fastqc/). To analyze miRNA expression profiles both miRDeep 2.0.0.5 [24] and miRExpress 2.0 [25] were used. Briefly, short reads were mapped to the human (UCSC hg19) genome allowing a minimum read length of 18, zero mismatches in the seed region and a maximum of five genomic loci. Known human miRNAs were identified and quantified based on miRBase Release 19 [26] entries. Using miRExpress known human miRNAs were identified from miRBase Release 19 with an alignment identity of 1% a tolerance range of four and a similarity threshold of 0.8 in the analysis. Differential expression analysis was performed separately for miRDeep and miRExpress using a negative binomial distribution in EdgeR [27]. Only miRNAs with at least one count per million in at least half of the samples analyzed were used in expression analysis and counts were normalized using the trimmed mean of M-values normalization method [27]. For comparisons of matched samples (i.e. ICC tumor versus distal histologically normal tissue from the same patient) a generalized linear model was employed, using the Cox-Reid profile-adjusted likelihood method for estimating dispersion [27]. For comparisons of tumor tissue to non-CCA normal tissue the quantile-adjusted conditional maximum likelihood method was employed using moderated tagwise dispersion [27]. Differentially expressed miRNAs were defined as having a Benjamini and Hochberg corrected p value of < 0.05.

### Quantitative real time PCR

cDNA was generated from 250 ng of purified plasma RNA using the miScript RT II kit (Qiagen) with heparinase co-treatment during the RT reaction as described [23]. qPCR analysis was performed using the miScript SYBR Green PCR Kit (Qiagen) on custom printed 96 well miScript miRNA arrays (SABiosciences). Selected miRNAs and normalization controls are shown in Additional file 1: Table S2. qPCR was performed on a BioRad iCycler iQ5 with an initial activation step of 95°C for 15 minutes followed by 40 cycles of 3-step cycling (Denaturation, 15 seconds at 94°C; Annealing, 30 seconds at 55°C; and Extension, 30 seconds at 70°C) followed by melt curve analysis for 81 cycles at 55°C and 20 second dwell time. Quantitation was performed using the ΔΔCt method [28]. Ct values were exported and analyzed using SABiosciences data analysis tools (http://pcrdataanalysis.sabiosciences.com/mirna). Samples were normalized using miR-103a, −15b, −16, −191, −22 as well as cel-miR-39-3p (*C. elegans* mimic spike-in control).

### Database accession

Microarray data was previously prepared according to MIAME standards and deposited in the GEO (Gene Expression Omnibus Database, National Center for Biotechnology Information, U.S. National Library of Medicine, Bethesda, MD) under accession number GSE53992. RNA sequence data have been submitted to the Sequence Read Archive (National Center for Biotechnology Information, U.S. National Library of Medicine, Bethesda, MD) under accession number PRJNA275105 (Sample submission pending).

## Results

### RNA of suitable concentration and purity were obtained from FFPE and plasma samples

Using Qiagen’s miRNeasy FFPE kit, sections of FFPE tumor tissue yielded purified RNA with 260/280 and 260/230 ratios of 2.0 and 1.9, respectively, indicating that it was pure, and of suitable quality for downstream applications [12]. RIN scores were between 2–3 for RNA purified from FFPE samples, indicating degradation of larger RNA species, but, as miRNAs exhibit greater robustness in FFPE tissue [29] and RIN values have negligible effect on miRNA results [30], the purified RNA was considered suitable for further analysis including RNA-seq. As plasma contains small quantities of miRNA/RNA [31] and, typically, the quantity of plasma available is limited, we have previously evaluated techniques and kits to optimize isolation and yield [23,32]. Initial cDNA synthesis reactions demonstrated inhibition of transcription by residual heparin (co-purified from plasma samples) and this was overcome by co-treatment of the RNA with Bacteroides heparinase I during reverse transcription, as previously described [32]. Subsequent cDNA derived from plasma RNA was then successfully analyzed by qPCR using customized miRNA plates coated with 85 CCA specific miRNAs.

### Illumina sequencing showed enrichment of miRNA species in RNA from FFPE samples

**Figure 1:**
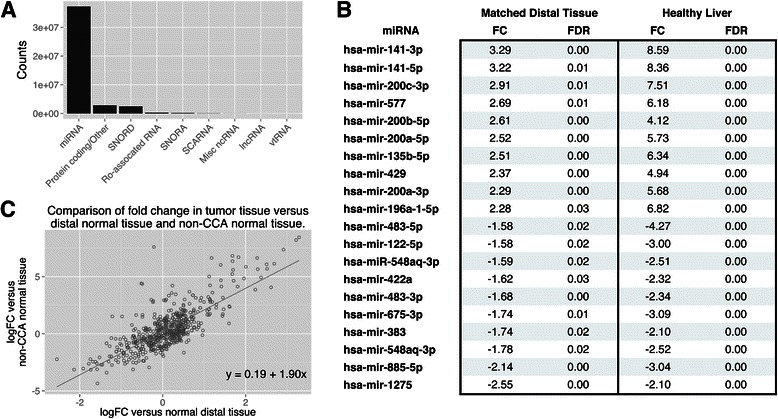
Summary of RNA-Seq analysis of CCA tumor tissue and controls. **A**. Mapping of short-reads to the human genome showed an enrichment of miRNA species versus protein coding genes and other small non-coding RNA species; **B**. Top ten significantly (BH corrected p < 0.05) up- and down-regulated miRNAs after differential expression analysis of tumor tissue and matched distal normal tissue. FC; Fold change, FDR; Benjamini and Hochberg corrected p value; **C**. Linear regression analysis (solid line) of miRNA fold changes in tumor tissue versus matched distal normal tissue (D-NT) and non-CCA normal liver tissue (N NT). Plot is annotated with the regression equation.

Using Illumina sequencing, the small RNA populations from the following samples were characterized: (1) ICC tumor tissue (CTT) (n = 14); (2) matched non-tumor tissue microdissected from the same ICC tumor block as the CTT but distal from observed dysplasia or frank carcinoma (D-NT; n = 14); and (3) normal liver tissue from biopsies of individuals undergoing gastric bypass surgery at George Washington University (N-NT; n = 9). Two different histological grades of Ov-induced ICC were represented in the sample set, well differentiated (n = 6) and papillary tumor (n = 8). Moderately differentiated FFPE were not analyzed in this study due to the lack of available tumor tissue. Approximately 246 million raw reads were obtained from these samples (∼10 million per sample) and, after quality filtering and short read removal, ∼143 million reads were retained. Before analysis with miRDeep, these reads were mapped to the human genome using Bowtie (−n 3 -l 28) and the reads successfully aligned ranged between 82—97% (average 85%). Using miRDeep, reads were compressed and remapped to the human genome and 86% of aligned reads mapped to miRNA genes (∼47 million reads), 6% to protein coding genes, and the remainder mapped to various small non-coding RNA species (Figure 1A). Counts were obtained for 690 miRNAs, each miRNA possessing greater than one count per million (cpm) in at least half of the samples. Analysis with miRExpress provided similar results with counts for 617 miRNAs obtained, each with greater than 1 cpm in at least half of the samples.

### ICC samples displayed a distinct profile of dysregulated tissue-based miRNAs

MicroRNA expression profiles of CTT were compared to their matched distal non-tumor tissue (D-NT). Using an additive linear model in EdgeR, 67 miRNAs were found to be significantly dysregulated when CTT were compared to D-NT, with 32 miRNAs significantly down-regulated and 35 significantly up-regulated (Figure 1B) (Benjamini and Hochberg (BH) corrected p value of < 0.05). The CTT expression profile was also compared to non-tumor tissue taken from control individuals (N-NT) and 316 miRNAs were called as significantly dysregulated (BH corrected p < 0.05); 144 significantly up-regulated and 172 significantly down-regulated (Figure 1B; Additional file 2: Table S1). The 316 significantly dysregulated miRNAs from the N-NT comparison included all but eight of the miRNAs identified as dysregulated when CTT tumor tissue was compared with D-NT tissue and all of these had the same direction of dysregulation.

### MicroRNA profiles from ICC tumor tissue displayed more similarity to distal tissue from the same block than with normal “non tumor” tissue

**Figure 2:**
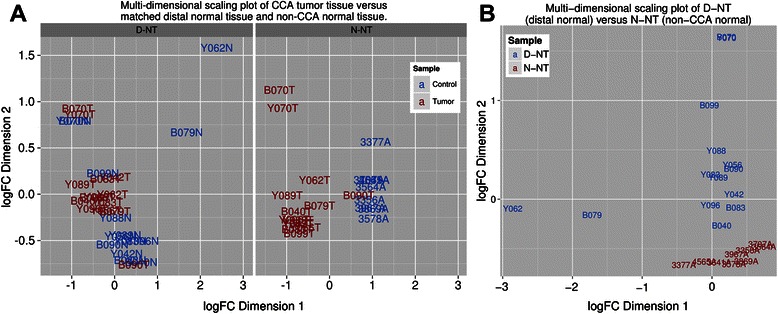
Multi-dimensional scaling plots comparing miRNA expression levels in different tissue. **A**. Multi-dimensional scaling plots comparing miRNA expression levels in CCA tissue versus matched distal normal tissue (Distal) and non-CCA normal liver tissue (Non-CCA). When compared to non-CCA normal tissue, tumor tissue grouped together but fewer differences where observed when comparing tumor tissue to its matched distal normal tissue. **B**. Comparison of miRNA expression in the two control samples, D-NT and N-NT. Multi-dimensional scaling plot of comparison between raw counts obtained from D-NT and N-NT. A clear differentiation between the two samples can be seen.

The pattern of miRNA dysregulation from CTT samples was similar when compared to both D-NT and N-NT controls. Linear regression analysis of fold change (FC) values from the two experiments gave an *R*2 value of 0.60 and a y-intercept of 0.19 (Figure 1C). However, the magnitude of the FC values for miRNAs found to be significantly dysregulated was greater when CTT was compared to N-NT than when CTT was compared to D-NT (Figure 1C). To visualize the grouping of test and control samples, multi-dimensional scaling (MDS) plots were used as shown in Figure 2. These plots generate distances between samples corresponding to the biological coefficient of variation between the most heterogeneous genes in each sample [27]. In MDS plots comparing CTT and N-NT, a distinct grouping of tumor and control tissue can be observed (Figure 2A, right). Conversely, MDS plots comparing CTT and D-NT showed no distinct grouping of tumor and control tissue (Figure 2A, left), suggesting fewer differences between these sample types. When D-NT and N-NT miRNA levels were directly compared, clear differences were observed: 200 miRNAs were significantly dysregulated, with 116 up-regulated and 84 down-regulated. The two types of control samples (D-NT and N-NT) clearly clustered into two distinct groups when compared in a MDS plot (Figure 2B).

### Papillary tumors exhibited greater miRNA dysregulation than well-differentiated tumors

**Figure 3:**
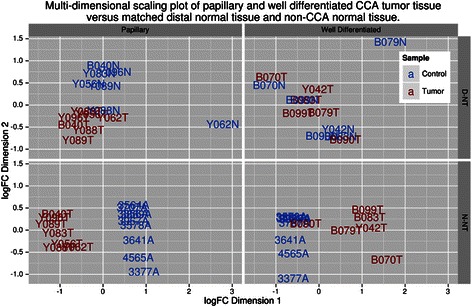
Multi-dimensional scaling plots comparing differently graded tumor tissue to matched normal distal tissue and non-CCA normal liver tissue. EdgeR [27] was used to measure distances between the miRNA expression profile of papillary and well differentiated tumor tissue to D-NT and N-NT. When compared to non-CCA normal liver tissue both papillary and well differentiated tumor samples were clearly distinguishable from the control samples. Conversely, when compared to matched D-NT only papillary samples were clearly distinguishable from the control samples.

Sufficient RNA was recovered from papillary ICC (n = 8) and well differentiated ICC (n = 6) samples to compare the effect of histological differentiation on miRNA profiles. No significantly dysregulated miRNAs were identified in well-differentiated tumor samples, when compared to D-NT. Conversely, 147 dysregulated miRNAs were identified in papillary tumors when compared to D-NT, with 78 up-regulated and 69 down-regulated (Additional file 2: Table S1). These included 64 of the 67 miRNAs found to be dysregulated when comparing all 14 tumor samples to D-NT controls. This can be observed visually in MDS plots, comparing papillary and well-differentiated tumor tissue to their matched D-NT tissue, with papillary tumor samples forming a distinct group versus the control groups. Well differentiated ICC did not form a unique group (Figure 3 top row). Both forms of tumor tissue grouped together when compared to N-NT (Figure 3 bottom row) and, once again, well differentiated tissue had fewer significantly dysregulated miRNAs (245) than papillary tissue (322). The majority of dysregulated miRNAs (71%) in the papillary tumors when compared to D-NT were also identified as dysregulated in the comparison with N-NT.

### Small RNA-Seq profiling of ICC tissue verified microarray profiling

In previous work [12], we comprehensively profiled these very same tumor tissue samples using the Agilent human miRNA microarray (miRBase Release 16.0). In comparison to Illumina sequencing, microarray analysis resulted in the identification of 28 (cf. 147 using NGS) and 120 (cf. 322 using NGS) dysregulated miRNAs in papillary tissue versus D-NT and N-NT controls respectively. Likewise, in well differentiated tissue 12 (cf. none using NGS) and 61 (cf. 245 using NGS) dysregulated miRNAs were identified. On both platforms a subset of 20, 15 and 49 common miRNAs were identified in comparisons of well differentiated tissue to N-NT, papillary tissue to D-NT and papillary tissue to N-NT, respectively (Additional file 1: Figure S1A). Previous studies have shown that statistical measures of significance can vary when analyzing differential expression by microarray versus NGS platforms [23,33]. Accordingly, FC values of significantly dysregulated miRNAs from the microarray study were compared to the FC values of the same miRNAs determined using RNA-Seq, with a strong association observed between the values (Pearson’s coefficient (PC) of 0.94; Additional file 3: Figure S1B). For papillary ICC tissue samples, there was a good correlation (PC = 0.97; Additional file 3: Figure S1B) for miRNAs significantly dysregulated using both discovery methods. Likewise, although no miRNAs were significantly dysregulated in the RNA-Seq of well-differentiated ICC, a comparison of the FC values determined by microarray and by RNA-Seq showed a reasonable association (PC = 0.63; Additional file 3: Figure S1B).

### PCR of matched plasma samples revealed a miRNA expression profile specific to ICC

Following the dysregulated miRNA identification pipeline from tissue-based discovery to verification in blood, eighty-five dysregulated miRNAs (Additional file 1: Table S2) were included on custom-made qPCR plates based on the significant dysregulation observed in both microarray analysis [12] and in small RNA-Seq profiling of the same Ov-induced ICC tumor tissue performed here. The custom printed PCR plates were used to screen plasma-isolated RNA paired with the Ov-induced ICC tissue samples used in microarray and small RNA-Seq. Four Ov-associated ICC plasma samples from patients with well differentiated ICC, two with moderately differentiated ICC, and six with papillary ICC were analyzed by qPCR. All samples were matched to the tissues analyzed using RNA-Seq, with the exception of the moderately differentiated samples (see Table 2). Five plasma controls for normalization were included, along with a C. elegans control (miRTC), and PCR controls for normalization and quality control (PPC) (Additional file 1: Table S2).

**Figure 4:**
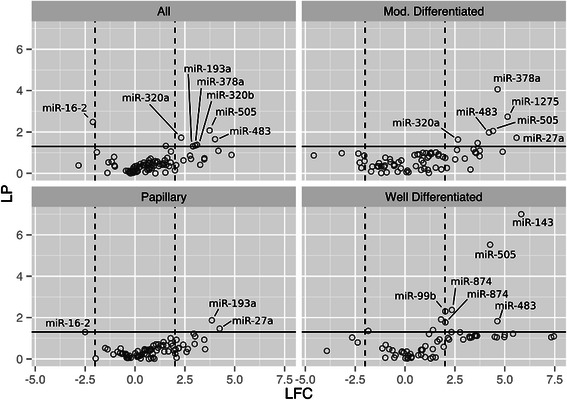
Circulating miRNA expression profiles determined using qPCR. Customized qPCR plates were used to profile 85 miRNAs dysregulated in CCA tumor tissue. Volcano plots show log fold change for each miRNA assayed versus log of the P value. Dotted lines represent 2-fold dysregulation and the solid line represents a p value of 0.05. Comparisons were made between all plasma from all CCA patients (All) and five non-endemic normal plasma control samples. Comparisons were also made between control samples and tumor samples grouped by the histological grading of the matched tumor sample.

**Figure 5:**
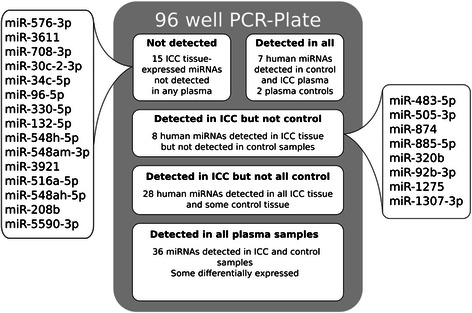
Summary of miRNAs detected during PCR analysis of plasma samples. Custom-made qPCR plates were used to profile 85 miRNAs found to be dysregulated in CCA tumor tissue. Fifteen miRNAs, highly dysregulated in tumor tissue, were not detected in any plasma samples and eight were detected in all ICC plasma samples but no controls. Thirty-six miRNAs were detected in all plasma samples, including those miRNAs found to be differentially expressed in ICC plasma.

**Figure 6:**
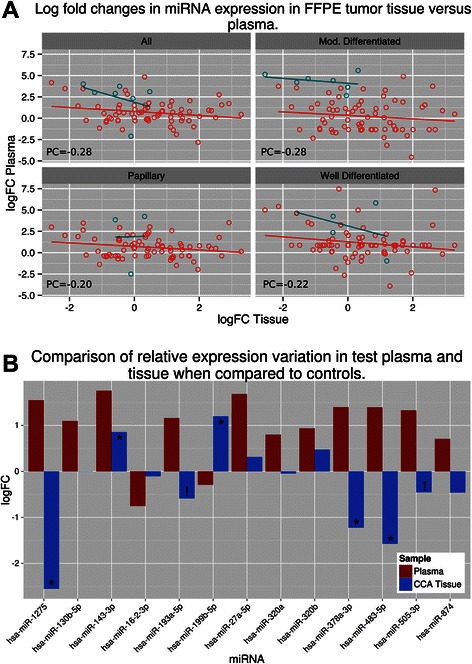
Log fold changes in miRNA expression in FFPE tumor tissue versus plasma. **A**. Scatter plots showing correlations between log fold changes (FC) in CCA tissue and matched tissue samples. A weak negative correlation was observed across all miRNAs assayed in qPCR experiments when compared with their FC in matched tissue samples. **B**. Comparison of miRNA FC in plasma and matched tissue samples in thirteen dysregulated miRNAs. Dysregulated miRNAs include those from all comparisons, including each of the histological grading comparisons. Of these thirteen miRNAs, seven exhibited inverse expression values between plasma and tissue. Asterisk denotes that the miRNA was observed to be significantly dysregulated in RNA-Seq experiments comparing all tumor samples to matched distal normal tissue and an exclamation mark denotes that the miRNA was found to be significantly dysregulated in the comparison of papillary tumor tissue with its matched distal normal sample.

When plasma from matched Ov-induced ICC samples, regardless of histology, were compared to control plasma, seven miRNAs were found to be dysregulated (Figure 4). When histology was considered, six, three and six miRNas were dysregulated in moderately differentiated, papillary and well differentiated ICC, respectively (Figure 4). Interestingly, the 15 most highly dysregulated miRNAs observed in the tissue-based discovery stage were absent in paired plasma samples (Figure 5, Additional file 4: Table S3). Accordingly, these 15 miRNAs appear to be dysregulated exclusively in tumor tissue. Moreover, while seven miRNAs were amplified in both case and control plasma, eight miRNAs were amplified exclusively in the ICC plasma but not in control plasma, suggesting a circulating miRNA profile exclusive to ICC (Figure 5, Additional file 4: Table S3). Surprisingly, only two of these 8 miRNAs were down-regulated in tissue using RNA-Seq. Indeed, there was a slight inverse ratio between expression levels of dysregulated miRNAs in tissue and plasma (PC between −0.20 and −0.28 for the differently graded tissue) (Figure 6). This was particularly evident in miRNAs significantly dysregulated in plasma samples. Thirteen miRNAs were dysregulated in at least one of the above comparisons and seven of these showed an inverse FC when compared to their expression in ICC (Figure 6B).

## Discussion

MicroRNAs have great potential as predictive, diagnostic and prognostic biomarkers for Ov-induced ICC, making an understanding of the ways in which miRNA expression levels vary during ICC tumor progression essential. This manuscript expands on our previous tissue-based miRNA discovery efforts by microarray (miRBase 16.0) by employing Next Generation Sequencing (small RNA-Seq) on the same sample set [12]. Here, we again observed that increasing histological differentiation of Ov-induced ICC tumors is reflected in an increasing number and magnitude of dysregulated miRNAs, suggesting that miRNA regulation is a key process in tumor differentiation. The use of small RNA-Seq also confirmed that adjacent non-tumor tissue (D-NT), which has with no dysplasia or frank carcinoma, shares similar miRNA dysregulation profiles with adjacent tumor tissue (CTT). Finally, our analysis of matched plasma samples by quantitative PCR showed than an eight-miRNA expression profile strongly associated with ICC.

Due to the location of ICC tumors in the upper hepatoduodenal ligament and the proximity of these tumors to the lymphatic and vascular systems of the liver [2], we expected ICC tumors to shed miRNAs into the blood stream, as observed with other solid tumors (e.g., metastatic breast, colon, and prostate cancers as reviewed in [19]). As Ov-induced ICC poses unique diagnostic and prognostic challenges, an accessible early diagnostic marker in blood is greatly needed. Towards this end, we generated a custom made qPCR plate containing miRNAs found to be dysregulated in ICC tumor tissue by small RNA-Seq to target these miRNAs in plasma matched samples. Eight of these dysregulated miRNAs in plasma emerged as strongly associated with ICC: i.e., eight dysregulated miRNAs were identified in all Ov-induced ICC plasma samples and not in control plasma (Figure 5). Interestingly, a negative correlation was observed between the expression levels of these eight miRNAs in tissue and in their matched plasma samples (Figure NA), with seven displaying opposite expression changes in plasma to that in tissue (miR-1275, miR-193a-5p, miR199b-5p, miR-320a, miR-483-5p, miR-505-3p, miR-874) (Figure NB). A similar inverse relationship between tissue and blood based miRNA dysregulation has been reported for several other cancers and pathologies, including for another infection-related cancer (nasopharyngeal carcinoma) by our own group [23], as well as breast cancer [34], endometrioid endometrial carcinoma [35], leukemia [36], neointimal hyperplasia [37] and also in atherosclerotic abdominal aortic aneurysm [38]. An additional 13 significantly dysregulated miRNAs were observed only when matched plasma was compared to control plasma, indicative of miRNA solely found circulating in the plasma of NPC cases not found in their tumor tissue. These results reflect on the possible different functions of miRNAs in tissue and circulating in peripheral blood. Moreover, the recent finding of circulating exosomes (or microvesicles) “laden” with miRNAs secreted from the bile duct of individuals with ICC offers intriguing possibilities for miRNA trafficking. As exosomes are actively exported from cells and incorporated into cells from the blood, they offer an explanation that cancer cells are able to selectively export or import particular miRNAs via these microvesicles, which would explain the inverse expression levels in tissue and plasma [36,39].

In this regard, the absence of a linear association between miRNA expression levels in tumor tissue and blood suggests that the primary focus of plasma biomarker discovery should be the plasma itself and not the primary tumor tissue, as we have previously assumed for our biomarker discovery pipeline [12,23]. The finding of divergent expression profiles in tumor tissue and matched plasma samples is especially intriguing for Ov-induced ICC, given the proximity of Ov-induced ICC tumors to the lymphatic and vascular systems of the liver [2]. In addition to the validation in a large sample set of potential miRNA biomarkers identified here, we plan to investigate the trafficking of miRNAs by exosomes in future studies. Moreover, multiple novel miRNAs (not in miRBase) were detected in the tissue samples examined by RNA-seq and we plan to validate the association of these miRNAs with ICC in plasma and tissue and determine whether they are human miRNAs and not contributed by Ov during infection.

Ov-induced ICC tumor tissue showed few differences from adjacent “non-tumor tissue” (D-NT) in miRNA expression profile (see the MDS plot in Figure 2A). However, when histological grade was taken into account, papillary ICC tumor did show significant differences compared to its adjacent non-tumor tissue D-NT, while well-differentiated tissue exhibited no differentiation with paired distal tissue (Figure 3). This suggests a regulation of different subsets of miRNAs during tumor progression, an observation consistent with findings in hepatocarcinoma [40,41], where differences in the composition, numbers and relative expression levels of miRNA increase with increasing histological differentiation. Functional studies also suggest that associations between the miRNA expression profile and histological grade are derived from miRNA regulation of key processes in tumor differentiation [40,41]. In this context, the differences observed in miRNA dysregulation between papillary and well differentiated tumor tissue in this study, likely reflect the fact that, by definition, well differentiated tumor tissue has the most resemblance to the bile duct tissue from which the tumor arose. Similarly, in comparisons of differently graded tumor with N-NT, well differentiated tissue exhibited fewer differences with the control tissue than papillary tissue. Indeed, in all comparisons using either D-NT or N-NT control tissue similar expression profiles were observed (as reflected in the PC of 0.60 between fold-change values generated using the two controls) but with greater magnitude of dysregulation in comparisons using N-NT. Tumors and their surrounding microenvironment are in constant interaction and the greater similarity between D-NT and CTT could reflect the influence of the tumor on surrounding tissue. Although, in this study, the difficultly in obtaining control tissue (discussed below) makes it difficult to ascertain the extent of such an effect.

We also tested different discovery platforms to identify signatures of miRNAs in Ov-induced ICC tumor FFPE tissue. Our previous approach [12] used a “targeted platform”, where known miRNAs were surveyed in ICC FFPE samples by a microarray built using miRBase 16 [26]. Here, we used an “untargeted” discovery approach, with a high throughput analysis of all small RNA species in case and control FFPE and found that the expression profiles determined by Illumina sequencing were similar to those determined in our microarray studies of Ov-induced ICC [12]. Robust correlations were observed between miRNA expression ratios obtained by microarray [12] with those obtained by Illumina miRNA sequencing (PCs of 0.97 and 0.63 for papillary and well differentiated tumors respectively as shown in Additional file 3: Figure S1). When comparing significantly dysregulated miRNAs, however, differences were observed between the two methods (Additional file 3: Figure S1A). In previous work comparing microarray and NGS analysis of miRNA expression levels, we [23], and others [33], have reported variations in the statistical assessment of significantly dysregulated miRNAs despite the overall similarity in fold change values. This may be due to cross-hybridization of closely related miRNA species on the microarray [23,33] or differences in the statistical methods employed by the two platforms, for example t-tests in microarray analysis and empirical Bayes estimation and exact tests based on the negative binomial distribution in NGS [27]. However, in the current manuscript the strong similar profiles obtained by these two discovery platforms suggests that the miRNA profiles reported here are an accurate representation of those for tissue-based miRNAs for Ov-induced ICC, despite differences in significance calling between the two platforms.

An obvious limitation of the current study was the lack of a predominantly cholangiocytic control tissue. As this type of sample is extremely rare, normal liver tissue (N-NT) obtained from liver biopsies of patients undergoing gastric bypass surgery was the best available control to represent liver tissue from non-Ov-induced ICC individuals. Nonetheless, the results reported here are, for the most part, in accord with literature (Table 1) suggesting not only that these results are an accurate reflection of the miRNA expression profile of Ov-induced ICC but that miRNAs reported here could have some utility in non-Ov-induced cholangiocarcinoma. Despite the limitations imposed by this control sample, it is clear that the miRNA profiles of D-NT tissue were more similar to ICC tumor tissue than to normal liver tissue N-NT. When ICC tumor tissue was compared to D-NT tissue, MDS plots showed differences in miRNA profiles when comparing tumor expression profiles to N-NT but not when compared to D-NT. Apart from intrinsic differences between the tumor tissue and N-NT, it is well documented that the tumor microenvironment is a major contributor to metastatic potential and the similarities between tumor tissue and their nearby non tumor tissue (D-NT) reflect this. Metastasis is closely associated with changes such as epithelial-mesenchymal transition, angiogenesis, matrix degradation, and stroma remodeling that occurs in the microenvironment [42]. A large number of miRNAs have been associated with metastasis (reviewed in [43]), at least one, miR-1 (also found to be dysregulated in this study), has been shown to directly influence the microenvironment of glioblastomas [44]. Another miRNA identified in our analysis, miR210, has been repeatedly implicated in the establishment of hypoxia [45-47]. Interestingly, in the work described here, miR-210 was significantly up-regulated in tumor tissue when compared to N-NT but not when compared to D-NT, suggesting a role for this miRNA in the establishment of a hypoxic microenvironment in Ov-induced ICC. Extracellular exosomal transport of miR-210 and possible uptake by endothelial cells has been shown in leukemic and metastatic cancer cells [48,49] and the results reported are consistent with the potential trafficking of miRNAs to areas adjacent to the tumor.

## Conclusions

In summary, this is the first comparative analysis using the latest available methods and matrices for the discovery of Ov-induced biomarkers for ICC. We show that optimized extraction protocols could produce sufficient RNA from FFPE and plasma for miRNA discovery and verification. While our study also showed the marked reproducibility between the two different miRNA discovery platforms (microarray and small RNA-Seq) when applied to FFPE, we concluded that RNA-Seq is the more informative method given its untargeted nature and the concomitant possibility of discovering novel miRNAs associated with a tumor. Third, and most intriguing, while the dysregulation profiles for subtypes of Ov-induced ICC tumors were not as strong in plasma as in matched tissue, eight miRNAs were identified only in case plasma and not control plasma, regardless of histology, as well as 13 dysregulated miRNAs detected solely in plasma. The results of this novel effort reflect the possible different functions of miRNAs for Ov-induced ICC in tissue and in peripheral blood and, more importantly, identify a candidate circulating miRNA profile that should be further explored as diagnostic biomarker in peripheral blood for Ov-induced ICC.

## Additional files

Additional file 1: Table S2. qPCR Plate Layout.
Additional file 2: Table S1. EdgeR output from miRNA differential expression analysis.
Additional file 3: Figure S1. Regression analysis comparing fold change (FC) values from miRNAs identified as significantly dysregulated in microarray analysis to FC values from RNA-Seq analysis of the same tissue. When all samples where compared to their matched D-NT tissue FC values were strongly correlated (Pearson’s correlation of 0.94; PC on graph). Similarly, when analyses were broken into comparisons between samples of the same histological grade, strong correlation was observed in the FC values obtained using both methods.
Additional file 4: Table S3. Summary of miRNA expression in plasma as measured using custom-made qPCR plate.
