# Supplementary material for: A microRNA profile associated with Opisthorchis viverrini-induced cholangiocarcinoma in tissue and plasma
Source: BMC Cancer. 2015 Apr 23;15:309. doi: 10.1186/s12885-015-1270-5 (PMC4417245; doi:10.1186/s12885-015-1270-5)
Supplement: Additional file 3: Figure S1. — Regression analysis comparing fold change (FC) values from miRNAs identified as significantly dysregulated in microarray analysis to FC values from RNA-Seq analysis of the same tissue. When all samples where compared to their matched D-NT tissue FC values were strongly correlated (Pearson’s correlation of 0.94; PC on graph). Similarly, when analyses were broken into comparisons between samples of the same histological grade, strong correlation was observed in the FC values obtained using both methods. [file 12885_2015_1270_MOESM3_ESM.pdf]

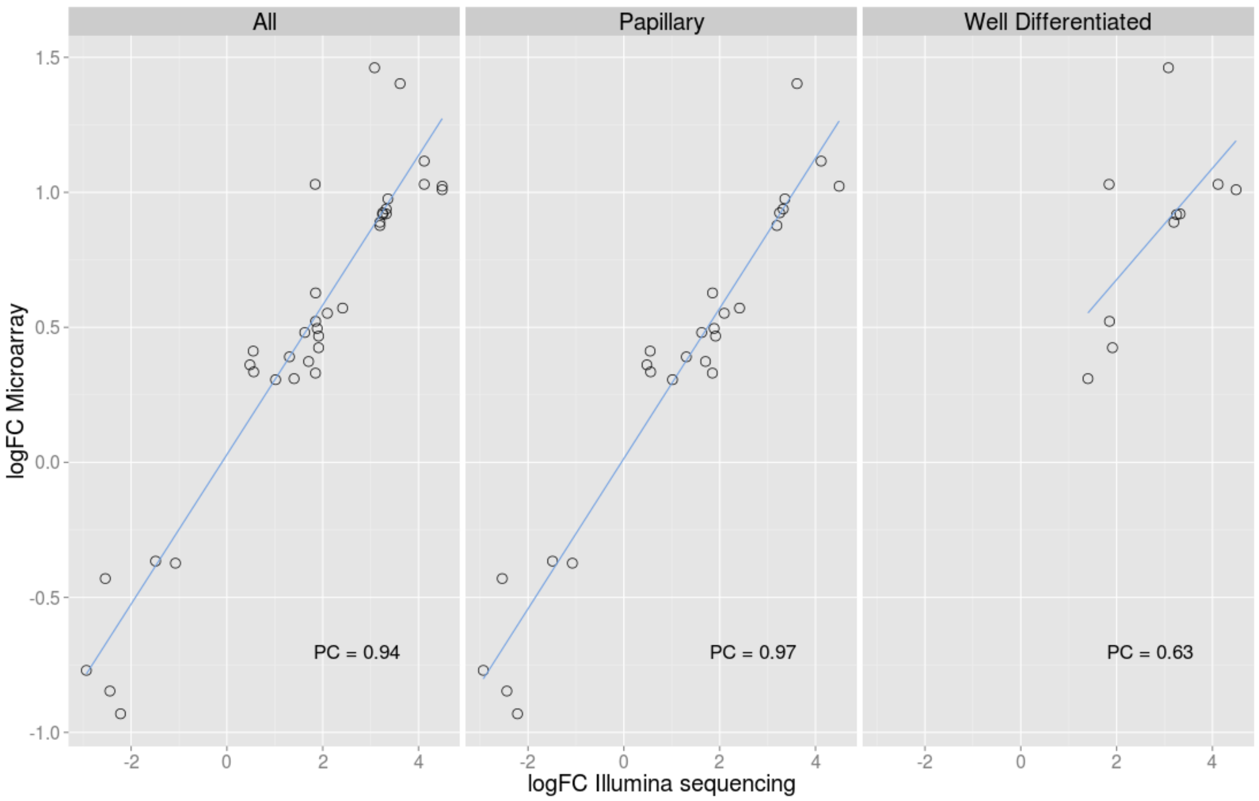

Supplementary Figure 1: Regression analysis comparing fold change (FC) values from miRNAs identified as significantly dysregulated in microarray analysis to FC values from RNA-Seq analysis of the same tissue. When all samples where compared to their matched D-NT tissue FC values were strongly correlated (Pearson's correlation of 0.94; PC on graph). Similarly, when analyses were broken into comparisons between samples of the same histological grade, strong correlation was observed in the FC values obtained using both methods.
